# Long-acting Cabotegravir/Rilpivirine for Treatment of HIV During Pregnancy: A Case Series

**DOI:** 10.1093/ofid/ofaf649

**Published:** 2025-10-22

**Authors:** Shawnalyn W Sunagawa, Carly Cloud Floyd, Sara H Bares, Anthony T Podany, Kimberly K Scarsi, Timothy Mykris, Lee Winchester, Jennifer O’Neill, Maureen Kubat, Christine Tran, Jennifer M Davis, Jeremy W Snyder, Joshua P Havens

**Affiliations:** Department of Pharmacy Practice and Science, College of Pharmacy, University of Nebraska Medical Center, Omaha, Nebraska, USA; Antiviral Pharmacology Laboratory, University of Nebraska Medical Center, Omaha, Nebraska, USA; Department of Pharmacy Practice and Science, College of Pharmacy, University of New Mexico, Albuquerque, New Mexico, USA; Division of Infectious Diseases, Department of Internal Medicine, College of Medicine, University of Nebraska Medical Center, Omaha, Nebraska, USA; Department of Pharmacy Practice and Science, College of Pharmacy, University of Nebraska Medical Center, Omaha, Nebraska, USA; Antiviral Pharmacology Laboratory, University of Nebraska Medical Center, Omaha, Nebraska, USA; Department of Pharmacy Practice and Science, College of Pharmacy, University of Nebraska Medical Center, Omaha, Nebraska, USA; Antiviral Pharmacology Laboratory, University of Nebraska Medical Center, Omaha, Nebraska, USA; Division of Infectious Diseases, Department of Internal Medicine, College of Medicine, University of Nebraska Medical Center, Omaha, Nebraska, USA; Antiviral Pharmacology Laboratory, University of Nebraska Medical Center, Omaha, Nebraska, USA; Antiviral Pharmacology Laboratory, University of Nebraska Medical Center, Omaha, Nebraska, USA; Division of Infectious Diseases, Department of Internal Medicine, College of Medicine, University of Nebraska Medical Center, Omaha, Nebraska, USA; Division of Infectious Diseases, Department of Internal Medicine, College of Medicine, University of Nebraska Medical Center, Omaha, Nebraska, USA; Division of Infectious Diseases, Department of Internal Medicine, College of Medicine, University of Nebraska Medical Center, Omaha, Nebraska, USA; Division of Infectious Diseases, Department of Internal Medicine, College of Medicine, University of Nebraska Medical Center, Omaha, Nebraska, USA; Division of General Internal Medicine, Department of Internal Medicine, School of Medicine, University of New Mexico, Albuquerque, New Mexico, USA; Department of Pharmacy Practice and Science, College of Pharmacy, University of Nebraska Medical Center, Omaha, Nebraska, USA; Division of Infectious Diseases, Department of Internal Medicine, College of Medicine, University of Nebraska Medical Center, Omaha, Nebraska, USA

**Keywords:** HIV, long-acting cabotegravir/rilpivirine, pregnancy

## Abstract

We present pharmacokinetic data on long-acting cabotegravir/rilpivirine (LA CAB/RPV) from two patients during their second and third trimesters. Monthly LA CAB/RPV dosing maintained concentrations above the proposed efficacy thresholds while bimonthly dosing resulted in subtherapeutic RPV concentrations. This highlights the potential of monthly LA CAB/RPV dosing in pregnancy.

## Teaser

In this case series, we present pharmacokinetic data from two pregnant patients on long-acting cabotegravir/rilpivirine during their second and third trimesters. Once monthly dosing maintained concentrations above the proposed efficacy thresholds, whereas bimonthly dosing led to subtherapeutic rilpivirine concentrations, highlighting the potential utilization of once monthly long-acting cabotegravir/rilpivirine during pregnancy.

## INTRODUCTION

Antiretroviral therapy (ART) adherence during and immediately after pregnancy is a significant challenge that must be addressed to eliminate perinatal/vertical HIV transmission [1]. Long-acting ART, specifically long-acting cabotegravir/rilpivirine (LA CAB/RPV), may help address adherence challenges in this population [2, 3]. However, there is limited published data surrounding utilization of LA CAB/RPV during pregnancy [4, 5]. Furthermore, LA CAB/RPV was discontinued in all who became pregnant in registrational clinical trials. Although there were no safety concerns, all pharmacokinetic data were obtained after participants had discontinued therapy, limiting our ability to assess whether these dosing strategies are appropriate for pregnant individuals [4, 6]. Here, we present two cases of LA CAB/RPV utilization and drug concentrations during pregnancy and the immediate postpartum period.

## CLINICAL CASES

**Figure 1.**
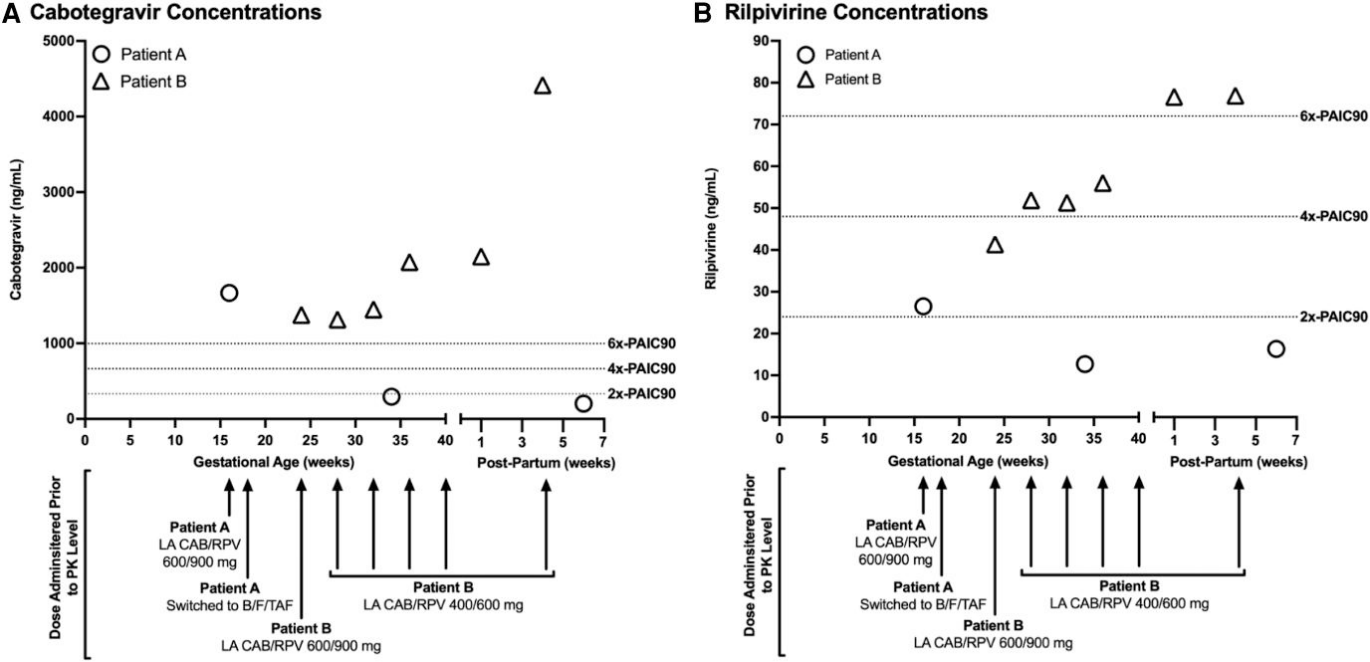
Antiretroviral, trough concentrations, and gestational/postpartum time course. Abbreviations: B/F/TAF, bictegravir/emtricitabine/tenofovir alafenamide; CAB, cabotegravir; RPV, rilpivirine; LA, long-acting; mL, milliliter; ng, nanogram; PAIC90, protein-adjusted 90% inhibitory concentration.

Patient A was a 24-year-old female with perinatal HIV with a body mass index (BMI) range during pregnancy of 26.4–34.2 kg/m^2^. She contacted the clinic due to a reported positive home pregnancy test with her last menstrual period ∼8 weeks prior. She had previously switched from bictegravir/emtricitabine/tenofovir alafenamide (B/F/TAF) to LA CAB/RPV 600/900 mg every other month (ie, bimonthly) injections ∼9 months prior and had remained virologically suppressed with no missed or late injections. Her ART history included efavirenz + lopinavir/ritonavir + stavudine, lopinavir/ritonavir + abacavir + lamivudine, atazanavir + ritonavir + emtricitabine/tenofovir disoproxil fumarate, darunavir + ritonavir + emtricitabine/tenofovir disoproxil fumarate, and darunavir/cobicistat + F/TAF. HIV RNA drawn at this visit was undetectable; no baseline or historical HIV drug resistance genotypes were available. She was instructed to return for a scheduled visit at ∼12 weeks’ gestation for potential ART adjustment and a repeat HIV RNA. At this visit, the multidisciplinary team (MDT), including HIV physicians, advanced practice providers, pharmacists, and nurses, discussed with her the option of transitioning back to B/F/TAF as LA CAB/RPV was not preferred during pregnancy [1]; however, Patient A voiced a strong desire to remain on LA CAB/RPV. The follow-up visit HIV RNA at 12 weeks’ gestation was also undetectable but given potential for subtherapeutic RPV concentrations during pregnancy [4, 7, 8], she was prescribed oral F/TAF daily to add to LA CAB/RPV and asked to return in 4 weeks for her scheduled bimonthly LA CAB/RPV 600/900 mg injection. At this visit, she was ∼16 weeks’ gestation (Figure 1) and still desired to remain on LA CAB/RPV. She noted significant nausea/vomiting that she attributed to the oral F/TAF and elected to stop oral ART prior to this clinic visit. The MDT obtained an HIV RNA along with trough CAB and RPV concentrations at this visit prior to administering LA CAB/RPV 600/900 mg. The CAB and RPV concentrations were 1666 ng/mL (CAB 4x-protein-adjusted 90% inhibitory concentration [PA-IC_90_] = 664 ng/mL) and 26.53 ng/mL (RPV 4x-PA- IC_90_ = 48 ng/mL), respectively, and HIV RNA remained undetectable (Figure 1) [9]. Two weeks later, these results were reviewed with Patient A and the shared decision was made to transition back to previously tolerated oral B/F/TAF daily instead of trialing monthly LA CAB/RPV 400/600 mg dosing since RPV concentrations were potentially subtherapeutic. Additional washout plasma CAB and RPV concentrations were obtained at 34 weeks’ gestation and 6 weeks postpartum (∼18 weeks and ∼30 weeks after final LA CAB/RPV dose, respectively) and are reported in the Figure 1.

Patient B, a 25-year-old female (BMI range during pregnancy: 29.4–34.2 kg/m^2^) living with HIV for 10 years presented for scheduled follow-up and reported two positive home pregnancy tests with last menstrual period ∼6 weeks earlier. Patient B was virologically suppressed on bimonthly LA CAB/RPV 600/900 mg for ∼16 months prior to the visit with no missed or late injections. ART history included darunavir + ritonavir + emtricitabine/tenofovir disoproxil fumarate, elvitegravir/cobicistat/emtricitabine/tenofovir alafenamide, and B/F/TAF. The MDT discussed switching to oral B/F/TAF, but Patient B desired to remain on LA CAB/RPV due to previous oral ART adherence challenges. HIV RNA drawn at this visit was undetectable; no baseline or historical HIV drug resistance genotypes were available, and she received her LA CAB/RPV 600/900 mg dose as scheduled two weeks later (estimated 8 weeks’ gestation). At 16 weeks’ gestation, the MDT and patient decided to continue bimonthly LA CAB/RPV 600/900 mg. However, at 24 weeks’ gestation, Patient B and MDT together chose to switch to monthly LA CAB/RPV 400/600 mg dosing due to concern for subtherapeutic RPV levels during the second and third trimesters of pregnancy. Plasma trough CAB and RPV concentrations were obtained 8 weeks after the last bimonthly LA CAB/RPV 600/900 mg dose, and with each monthly LA CAB/RPV 400/600 mg injection from 24 weeks gestation through delivery and 4 weeks postpartum. CAB and RPV concentrations ranged from 1314–4416 ng/mL to 41.32–76.9 ng/mL, respectively (Figure 1). At the patient's 4-week postpartum visit, she transitioned back to bimonthly LA CAB/RPV 600/900 mg dosing for schedule convenience.

Both patients remained virologically suppressed throughout their entire pregnancy and delivered without any complications or adverse effects of pregnancy (eg, pre-eclampsia, preterm delivery). Additionally, both infants had no congenital abnormalities at birth. Given both parents were virologically suppressed at time of delivery, both infants received 2 weeks of zidovudine prophylaxis [1] and infant HIV RNA tests were negative at 4 and 6 weeks postpartum for both Infant B and Infant A, respectively. Both infants’ HIV RNA tests remained negative through 4 months after delivery.

## DISCUSSION

These cases provide a unique real-world pharmacokinetic perspective on LA CAB/RPV in pregnancy and the postpartum period. They underscore the ongoing need to better characterize the pharmacokinetics of LA ART in pregnancy to inform evidence-based clinical decision-making—particularly as interest in and preference for LA ART is high in women of reproductive age [10]. Both cases demonstrate the complexities of balancing patient autonomy with limited clinical evidence for utilization of LA CAB/RPV during pregnancy.

The CAB concentrations in both patients were comparable to those in nonpregnant individuals, which is also aligned with current population-based pharmacokinetic modeling and clinical pharmacokinetic data of LA CAB in pregnancy [4, 6, 7]. For Patient A, while CAB concentrations were above the proposed 4x-PA-IC_90_ efficacy threshold of 664 ng/mL, there was concern for having only one drug with sufficient drug concentrations since the RPV concentration was below the proposed 4x-PA-IC_90_ efficacy threshold of 48 ng/mL (Figure 1) [9]. Based on this RPV level and previous data describing lower RPV plasma concentrations in pregnant people, especially in the second and third trimesters, she was transitioned back to oral B/F/TAF daily with intensive MDT adherence counseling [1, 8, 11]. This case, along with the only other published report of LA CAB/RPV in pregnancy, demonstrated that bimonthly dosing results in lower RPV concentrations—suggesting that bimonthly dosing may not be viable during pregnancy [8].

Patient B, by contrast, initiated monthly LA CAB/RPV at 24 weeks’ gestation and continued this through 4 weeks postpartum. Unlike Patient A, all of Patient B's CAB and RPV concentrations remained above the proposed 4x-PA-IC_90_ [9] while she received monthly injections. The initial RPV trough concentration at 24 weeks’ gestation was below the proposed 4x-PA-IC_90_ for RPV [9] (Figure 1); however, this trough was 8 weeks after Patient B's last bimonthly LA CAB/RPV 600/900 mg. This low RPV trough concentration, in addition to population-based pharmacokinetic modeling, supported switching to monthly LA CAB/RPV dosing [7]. Although no clinical, real-world pharmacokinetic data on monthly LA CAB/RPV use during pregnancy have been published to date, population-based pharmacokinetic modeling supports our findings [7]. These models predict that bimonthly LA CAB/RPV dosing during the third trimester results in RPV concentrations falling below the proposed 4x-PA-IC_90_ efficacy threshold, whereas all monthly dosing simulations maintain concentrations above this threshold [7].

These patient cases emphasize several important clinical considerations when deciding to continue LA CAB/RPV throughout pregnancy. First, they represent a shared decision-making opportunity where risks and benefits should be discussed, especially in the context of LA ART improving adherence and thereby decreasing risk of vertical HIV transmission [2, 12]. Decisions must be carefully balanced with the limited available clinical data supporting the safety of LA CAB/RPV in pregnancy. From registrational trials and the Antiretroviral Pregnancy Registry, while LA CAB/RPV appears to be safe during fetal development, with no evidence of associated congenital abnormalities at birth, there is placental transfer of both CAB and RPV, which warrants further monitoring and research for exposed infants [5, 8, 13]. Furthermore, while pharmacokinetic data from Patient B support the potential viability of monthly LA CAB/RPV dosing throughout pregnancy; it also emphasizes the need for close monitoring. This includes the potential utility of therapeutic drug monitoring given significant intraindividual and interindividual variability of up to ∼30%–50% in CAB and RPV concentrations which may be further exacerbated by the individual physiologic changes during pregnancy [14–16].

The drug concentrations presented in this case series reveal that despite physiologic changes during pregnancy that may impact drug concentrations, transitioning to LA CAB/RPV 400/600 mg monthly throughout the second and third trimesters of pregnancy maintained plasma trough concentrations of CAB and RPV above the proposed efficacy thresholds in patients who initiated LA CAB/RPV prior to pregnancy. Additionally, the low RPV concentrations seen in both patients during bimonthly LA CAB/RPV 600/900 mg dosing demonstrate that bimonthly dosing may not be a reliable option for patients during their second and third trimesters of pregnancy. Finally, while this case series adds to the limited clinical pharmacokinetic data of LA CAB/RPV in pregnancy, future and ongoing prospective trials [17] and registries are needed to evaluate optimal dosing strategies and pharmacokinetics of LA ART across trimesters and the postpartum period to guide clinicians in making individualized, evidence-based recommendations for pregnant people receiving ART [18].
